# An Update on Neuroaging on Earth and in Spaceflight

**DOI:** 10.3390/ijms26041738

**Published:** 2025-02-18

**Authors:** Nik V. Kuznetsov, Yauhen Statsenko, Milos Ljubisavljevic

**Affiliations:** 1 ASPIRE Precision Medicine Research Institute Abu Dhabi, United Arab Emirates University, Al Ain P.O. Box 15551, United Arab Emirates; e.a.statsenko@uaeu.ac.ae (Y.S.); milos@uaeu.ac.ae (M.L.); 2 Department of Radiology, College of Medicine and Health Sciences, United Arab Emirates University, Al Ain P.O. Box 15551, United Arab Emirates; 3 Department of Physiology, College of Medicine and Health Sciences, United Arab Emirates University, Al Ain P.O. Box 15551, United Arab Emirates

**Keywords:** brain aging, neuroaging, neurodegeneration, accelerated aging, space exposome, space motion sickness, spaceflight, Alzheimer’s disease, Parkinson’s disease, age-related diseases, biomarkers, non-coding RNAs

## Abstract

Over 400 articles on the pathophysiology of brain aging, neuroaging, and neurodegeneration were reviewed, with a focus on epigenetic mechanisms and numerous non-coding RNAs. In particular, this review the accent is on microRNAs, the discovery of whose pivotal role in gene regulation was recognized by the 2024 Nobel Prize in Physiology or Medicine. Aging is not a gradual process that can be easily modeled and described. Instead, multiple temporal processes occur during aging, and they can lead to mosaic changes that are not uniform in pace. The rate of change depends on a combination of external and internal factors and can be boosted in accelerated aging. The rate can decrease in decelerated aging due to individual structural and functional reserves created by cognitive, physical training, or pharmacological interventions. Neuroaging can be caused by genetic changes, epigenetic modifications, oxidative stress, inflammation, lifestyle, and environmental factors, which are especially noticeable in space environments where adaptive changes can trigger aging-like processes. Numerous candidate molecular biomarkers specific to neuroaging need to be validated to develop diagnostics and countermeasures.

## 1. Introduction

Traditionally, both the passage of time and aging have been viewed as uniformly occurring processes, independent of any conditions and circumstances. Accordingly, the rate of aging was assumed to be constant over time. In the last century, physics has undergone fundamental changes, providing a new universal understanding of the unification of space and time into space–time. As suggested by theories of relativity, the speed of time differs on the ground and in low Earth orbit (LEO), which has a number of practical applications, such as the need to adjust GPS satellite clocks daily by subtracting 38 microseconds [1].

**Figure 1:**
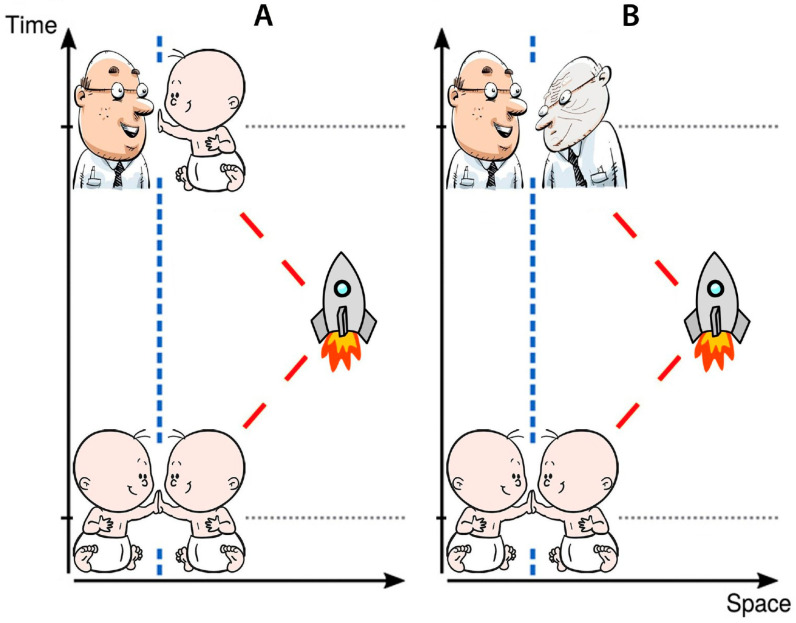
**Aging scenarios of future interstellar space travel** (see text for details): (**A**) Relativistic Twin Paradox scenario; (**B**) more realistic scenario.

In 1888 and 1895, H. Wells’ fiction novels introduced the idea of traveling in time. In 1911, physicists formulated the Twin Paradox that took hold of minds since it illustrated time dilation and decelerated aging. In this paradox, one of two twins takes a spaceflight at near-light speed to a distant star and later returns to Earth. Upon his return, he will be younger than his terrestrial twin (Figure 1A) according to Einstein’s special relativity [2].

However, biomedical observations have not supported the idea that spaceflight can slow down the aging processes; in fact, it has been found to accelerate aging (Figure 1B) [3,4,5,6,7,8,9,10,11,12,13,14,15,16].

*In vitro* models [17,18,19,20] and *in vivo* simulators [21,22,23] showed that artificial microgravity (MG) also revealed the phenomena of accelerated aging (AA). These results suggest the need for further research into the effects of AA and its prevention [24,25,26,27,28]. The necessity of countermeasures and the prophylactics of AA in future space missions serves as motivation for further studies.

The process of aging is linked to alterations in both structure and function, leading to heightened susceptibility to diseases and mortality [29,30,31]. Various factors contribute to aging including the accumulation of damage due to mutations, epigenetic changes, oxidative stress, and inflammation [32,33]. Physiological or biological age (BA) is defined as the current state of an individual as a biological system. The state of such a system is determined by a combination of biological parameters that affect life expectancy. These parameters include the current profile of genomic DNA methylation, age-associated structural changes in the brain, metabolic parameters, etc.

In normal aging (NA), BA is equal to chronological age. If aging is accelerated, as in cases of pathological aging, then biological age surpasses chronological age. In decelerated aging, BA becomes lower than official age [34,35,36]. While AA shares common features with NA, AA stands out due to distinct characteristics like protein aggregation and excitotoxicity that are exclusive to pathological aging [37,38,39]. Understanding mechanisms of aging opens opportunities for targeted treatment of the diseases that occur late in life [37].

AA represents a research field characterized by lingering challenges, including inconsistent terminology and poorly understood mechanisms [40,41]. Researchers have not reached an agreement on whether neurodegeneration (ND) is a type of AA or its outcome.

An alternative view suggests that particular biomarkers (BMs) are specific to ND and do not recognize AA [42,43,44,45,46]. In practice, no diagnostic BMs can prognosticate AA reliably [47,48,49,50,51,52,53,54,55,56,57,58].

Certain discrepancies between AA theories need to be addressed. Some hold that senile plaques are common neuropathological features in both healthy aging and ND, and that cerebral amyloid deposition is not necessarily associated with clinically apparent cognitive dysfunction. The development of cognitive deterioration requires additional factors, such as neuronal or synaptic loss or widespread cytoskeletal aberrations [59]. According to these studies, mesial and inferior temporal lobe structures are quite often affected by the formation of neurofibrillary tangles in the NA of the brain [60]. Other authors have presented evidence to support the opposite theory, suggesting that the early pathological changes associated with the disease represent the onset of ND and cerebrovascular disease rather than normal concomitants of aging [61]. In this way, neurofibrillary tangle formation may precede the emergence of the neuropsychological deficits typical of Alzheimer’s disease (AD) [60,61]; therefore, the understanding of the sequence of brain aging processes and their rates can be updated.

## 2. Objectives

The main objectives of this review are as follows:

- to pinpoint several groups of promising molecular biomarkers of aging with a special emphasis on various non-coding RNAs (Section 3);
- to draw a parallel between aging in spaceflight and on Earth and to consider the rates of aging through the lens of space biomedicine (Section 4);
- to discuss the applicability of the AA concept in the field of aging neuroscience, taking into consideration its limitations (Section 5);
- to outline a roadmap for the future of aging neuroscience (Section 6).

## 3. Biomarkers of Aging

Fields of research on aging and ND are closely related, as aging is the primary risk for the development of brain ND, especially AD; however, these diseases are not part of normal aging. Many theories claim to explain the etiology of AD (Table 1), including neurocentric and neurovascular hypotheses. At first, research was primarily concentrated on neurons. Later, the importance of non-neural cells in higher brain functions was recognized. Particularly, the hypothesis refers to a neurovascular unit (NVU), which is a dynamic multicellular structure mediating functional interactions between blood vessels and brain tissues [62]. The neural cells in an NVU and circulating immune cells secrete pro-inflammatory mediators contributing to inflammaging and endothelial disfunction. These changes disrupt molecular networks, induce BBB damage, and lead to NVU degeneration [63,64,65,66,67,68,69]. However, the exact role of an NVU in ND remains to be elucidated, and reliable ND-associated biomarkers to be found, due to the puzzling complexity of the NVU signaling and metabolic pathways [70,71,72,73].

Aging molecular biomarkers (MBMs) are biomolecules or their derivatives characterized by measurable parameters that can be used to estimate the progression of aging [97]. Aging MBMs include mRNA transcripts, proteins [98], telomere length, serum marker molecules [99], DNA methylation parameters [100,101], modifications of histones [102,103,104,105,106,107,108,109,110,111,112,113], differentially expressed genes [73,114], or non-coding RNAs [115,116,117,118].

Recent research has questioned whether age affects different cell types in NVUs. ON study resulted in the following candidate biomarker genes related to AA (AAGs): *IGF1R*, *MXI1*, *RB1*, *PPARA*, *NFE2L2*, *STAT5B*, *FOS*, *PRKCD*, *YWHAZ*, *HTT*, *MAPK9*, *HSPA9*, *SDHC*, *PRKDC*, and *PDPK1.* However, the differential expression of *IGF1R*, *MXI1*, *PPARA*, *YWHAZ*, and *MAPK9* correlated with ND progression, and it was not possible to justify AAGs as MBMs due to an insufficient sample size [73].

ND is a consequence of various structural alterations occurring across distinct genetic sites over a span of time [119,120]. A high risk of developing AD is associated with alterations in certain genes that predispose to ND (NDGs): *GBA1*, *APP*, *PSEN1*, *MAPT*, *GRN*, *SETX*, *SPAST*, *CSF1R*, *C9orf72* [121], *TET2* [122], *TBK1* [123], *TOMM40*, *APOC1* [124], *APOE* [124,125], and *TREM2* [126,127,128,129,130,131,132]. Nevertheless, the gene sets do not overlap across the studies on AAGs and NDGs. In addition, the *APOE* e4 allele and mutation spectrum for *TREM2* gene were found to be the risk factors for developing dementia with Lewy bodies, multi-cognitive decline, and corticobasal degeneration [132,133,134,135,136,137,138,139,140,141].

*DNA methylation* level reflects the rate of aging. Approximately 1.5% of genomic DNA contains 5-methylcytosine (5-mC), and this level decreases during ontogenesis [142]. The level of 5-mC is the highest in embryos, and then it reduces gradually across life [143,144]. In aging, global genomic DNA hypomethylation proceeds along with the hypermethylation of CpG islands (“epigenetic drift”), where 60% of them are associated with gene promoters and transcriptional regulome [145,146]. In NA, age-predictive models demonstrate gradual linear changes in the DNA methylation profile, but environmental or genetic risk factors can accelerate aging [147]. In monozygotic twins, the divergence of methylome increases at different rates [148]. Change in the DNA methylation profile was proposed as a mechanism of an epigenetic clock [149,150,151] by analogy with a biological clock [152,153]. Monitoring deviation between biological and chronological age helps to study development and aging across a lifespan [154]. The Horvath [155], Hannum [147], and PhenoAge [156] epigenetic clocks serve as markers of ND [156,157,158,159,160], with the first of these showing the strongest correlation between epigenetic and chronological age [160].

*Histone modifications* can serve as potential MBMs of aging, however, the heterogeneity of animal models used to develop the biomarkers limits their applicability. For example, a drop in highly abundant transcription activation mark H3K4me3 [102] correlated with an extended lifespan in *Caenorhabditis elegans* [103]. Contrarily, an increase in the H3K4me3 level was linked with AA in *Drosophila melanogaster* [104]. The level of heterochromatin-associated histone transcription repression mark H3K9me3 gradually decreases during aging in hematopoietic stem cells of humans and mice [105]. In *C. elegans* and other models of senescence, the most significant loss of H3K9me3 occurs in repressive regions [106,107]. H3K27me3 is associated with transcriptional silencing in aging [108]. The role of H3K27me3 is controversial, as studies have shown its bidirectional lifelong changes [109,110,111,112,113]. Increased levels of H4K20me3 and H3K4me3 and decreased levels of H3K9me1 and H3K27me3 are common age-associated epigenetic marks [161,162,163]. Research showed an increase in H3K4me3 promoter methylation in a CK-p25 tauopathy mouse model and the hippocampus of AD patients [164,165]. The following histone methylation marks can also be found in the brains of patients with Alzheimer’s: H4K20me2, H3K4me2, H3K27me3, H3K79me1, H3K79me2, H3K36me2, H4K20me3, H3K27me1, and H3K56me1 [166,167]. In addition, histone acetylation marks H3K9ac, H3K14ac, and H4K16ac are associated with normal and accelerated aging [163,164,166,167,168]. Histone phosphorylation marks H4S47p and H3S10p and histone ubiquitination mark H2BK120ub are observed in AD [167,169,170]. Further systematic research should elucidate the regulatory mechanisms of histone modifications, their interaction, and the interplay between histone marks and other factors.

*Non-coding RNAs* (ncRNAs) could be used as aging MBMs (Table 2) [171,172,173,174,175,176,177,178,179,180,181,182,183,184,185,186,187,188,189,190,191,192,193,194,195,196,197,198,199,200,201,202,203,204].

*Long non-coding RNAs* (lncRNAs), e.g., growth-arrest-specific transcript 5 (GAS5), play a significant role in cell proliferation and apoptosis [205,206,207]. Their down-regulation leads to phosphorylation of the tau protein in ND [208,209]. Long intergenic brain cytoplasmic RNA 1 (BCYRN1), expressed in the dendritic domains of neurons, is down-regulated in aging [198].

*MicroRNAs* (miRNAs) impact neuronal plasticity, influence tau protein metabolism, and mediate brain aging through the regulation of gene expression [210,211,212,213,214,215,216,217,218,219]. The regulation of miR-145a and miR-375 depends on age in mouse brains [183,220,221]. The MIR29 family, MIR339-5p, MIR195, and MIR107 modulate the expression of beta-secretase 1, involved in cleaving the amyloid precursor protein [181,222,223,224,225,226]. Interestingly, miR-34 plays a protective role in *Drosophila* [187], and MIR144/MIR451 regulates the ADAM metallopeptidase domain 10 in AD [227]. More than 20 miRNAs are secreted into cerebrospinal fluid by hypothalamic stem cells. These miRNAs control the aging rate in mice [186], which should also be relevant to the human brain [228]. Future studies should verify miRNAs as MBMs in humans [229].

*Circular RNAs (circRNAs)* are abundant in the brain, and their expression changes with age in skeletal muscles [230,231]. CircRNAs contribute to ND via interactions with miRNAs. For example, ciRS-7 potentially functions as a sponge for MIR7-1 [232], and its level is reduced dramatically in the brains of patients with AD [191]. Cerebral circRNAs are linked with neuronal maturation, neuroplasticity, and neurotransmitter and synaptic activities. They target specific age-related mRNAs in the brain affecting their expression and availability. At least four circRNAs are involved in postoperative neurocognitive disorders [193]. Another study revealed nearly 1200 cerebral circRNAs in a rat model of aging [192]. Various ncRNA biomarker candidates await validation in the clinical arena [194].

## 4. Accelerated Aging in Space

The space environment affects various organs and systems, causing different, sometimes unpredictable, rates of change. Space conditions induce changes similar to age-related changes on Earth: noticeable alterations in the structure and functioning of the brain [233,234,235,236,237,238,239,240] as well as loss of bone mass, muscle atrophy, and immune system impairment [241,242,243,244,245,246,247,248,249]. However, these deteriorations can occur in space at a fairly fast pace. It is becoming increasingly clear that the rate of brain aging may be influenced by space exposome factors including MG, exposure to radiation, intense workload, circadian rhythm perturbation, isolation, and confinement [250,251].

In particular, spaceflight appears to accelerate brain aging. The potential for cognitive impairment and cognitive changes commonly associated with aging, such as inflammatory responses, changes in brain metabolism, depression, and memory impairment during deep space missions, is a serious concern [233]. In spaceflight, like in aging, volumetric gray matter decreases [234,235] and changes occur in white matter [236,237,238,239]. Furthermore, in spaceflight, like in age-related declines, the deteriorations of the condition are partially offset by concomitant neuroplastic and neural compensatory processes [240].

Similarly, exposure to MG causes sarcopenia, a syndrome characterized by the loss of muscle mass and strength due to skeletal muscle unloading, resulting in senile phenotypes similar to those observed in older humans on Earth. Muscle atrophy is one of the most critical aging-like side effects of MG and a common problem in the geriatric population [252]. As expected, NASA had concerns about the inability of astronauts to perform normal everyday tasks in MG conditions [253]. One of the consequences, the common problem of orthostatic intolerance, has been observed in both astronauts and hospitalized elderly patients [254].

Likewise, bone cells respond and adapt to altered-gravity conditions by changing their morphology and function. Microgravity-associated bone density loss is due to an imbalance in bone remodeling caused by changes in osteoblasts, osteoclasts, osteocytes, and mesenchymal stem cells [255]. Bone loss in astronauts during spaceflight is a risk factor for premature osteoporosis, fractures, and kidney stones, and intense strength training cannot completely inhibit the increase in bone resorption biomarkers [256]. A recent examination of fractures recorded in the medical histories of all astronauts indicated a higher occurrence of hip and spine fractures among astronauts after long-term space missions in comparison to shorter spaceflights [257]. The comparison of quantitative CT-derived femoral trabecular bone loss in long-duration-spaceflight astronauts with terrestrial cohorts suggests that accelerated rates of trabecular bone mineral density loss during spaceflight are comparable with accelerated skeletal loss rates in aging women during menopause [257].

Last not least, chronic activation of the immune system, inflammaging, and immunosenescence are major contributing factors to several age-related pathologies [258,259,260]. Prolonged exposure to the space exposome may trigger maladaptation responses and promote chronic inflammation and stress responses, thus affecting various organ systems, exacerbating inflammaging, and inducing AA, which is a great apprehension for future spaceflights [261].

As part of the adaptation or stress response to the space exposome, various ncRNAs were identified (Table 3) [262,263,264,265,266,267,268,269,270,271,272,273,274,275,276,277], which makes them attractive as candidate biomarkers or potential points of pharmaceutical intervention in the development of countermeasures to space exposure. It remains to be seen whether these ncRNA-associated effects are specific to the response to cosmic factors or are also involved in aging on Earth.

The space exposome appears to act as an accelerator of biological aging, showing multiple interconnections between the biological aspects of spaceflight and the hallmarks of aging [278]. Space travel presents extraordinary circumstances and neurological hazards, caused by microgravity and exposure to space radiation. In a weightless environment, the vestibular system is affected, leading to issues like spatial disorientation, sensorimotor deficits, and space motion sickness (SMS). There are worries about increased risks of ND conditions such as AD and PD, as well as accelerated cognitive decline resembling premature aging. To address these challenges, it is crucial to develop further the countermeasures: pharmacological agents, diagnostics, and protective shielding from radiation [279].

## 5. Concept of Accelerated Aging

**Figure 2:**
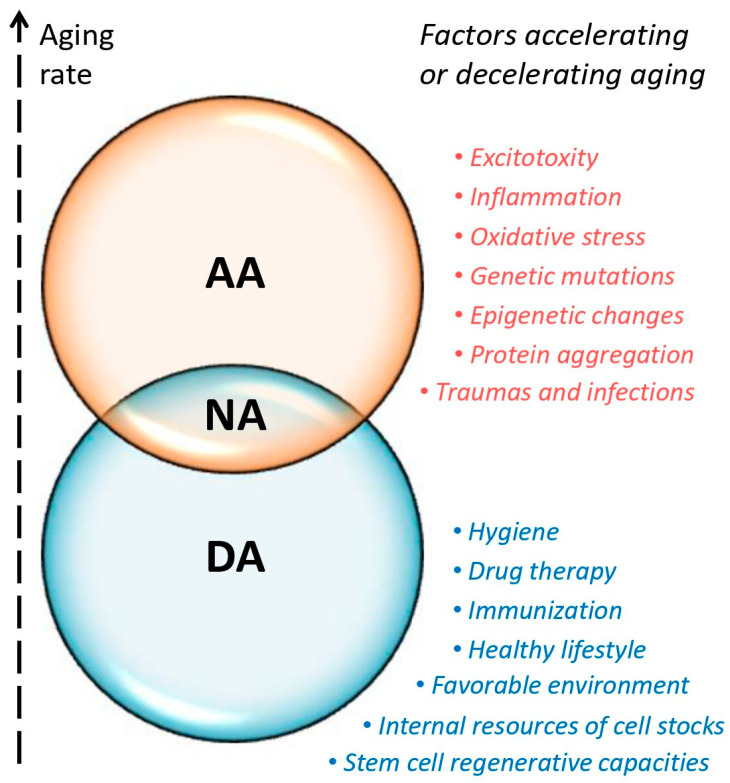
**Factors influencing the aging rate**. AA—accelerated aging; DA—decelerated aging; NA—normal aging.

NA can be defined as a conditional balance between AA and DA with a compromise combination of relevant factors (see Figure 2). Factors that accelerate aging include excitotoxicity, inflammation, oxidative stress, genetic mutations, epigenetic changes, protein aggregation, traumas, and infections. The factors decelerating aging are a healthy lifestyle, a favorable environment, hygiene, immunization, the regenerative capacities of stem cells, internal resources of cell stocks, and some types of drug therapy.

The AA concept should be considered within the context of individual capacities and personalized structure–functional reserve mechanisms. Aging and diseases lead to atrophy due to a reduction in the number of cells and supracellular structures [280]. The physiological reserves of an organ can be characterized as its total residual functional potential. In the context of brain aging, the physiological cognitive reserve reflects the level of education, occupational and environmental attainments, and performance in cognitive tests [280]. Reversible forms of mild cognitive impairment (MCI) and dementia represent clinical examples of restoring individual reserve potential [281,282]. Neural compensation in the elderly leads to the formation of secondary brain networks [283], which decelerate the aging of the brain [280,284]. In elderly patients, the reversal of MCI results from specific lifestyle activities and cognitive stimulation throughout life [285,286].

Age assessment requires an accurate estimation of multiple parameters that account for biological and chronological age differences. In neuroscience, machine learning models forecasted a lifetime spent in good health using brain-imaging data, with an error margin of 2.1 to 4.9 years [287,288].

Individual brain age can also be calculated as the difference between chronological age and predicted BA [289]. Overall BA depends on the reserve capacities of individual systems and organs [290,291]. The criteria for assessing AA of the brain are uncertain due to the absence of clear indicators for NA [292]. Methods for BA assessment are not standardized, and methodological discrepancies lead to contradictory findings in different studies. For example, AD adds 1.5 years to the brain age, MCI adds 1.0 years, multiple sclerosis adds 0.41 years, Parkinson’s disease (PD) adds 3 years, and schizophrenia adds 5.5 years. The cognitive impact of the last two pathologies is less severe and progresses at a slower pace compared to AD [293,294,295]. Another study on AD uncovered brain age additions ranging from 6 to 9 years [296].

In some cases, it is necessary to take into account certain methodological limitations. The studies on age-related brain atrophy commonly have a cross-sectional design that is less accurate compared to a longitudinal one [297]. Several studies are based on small non-representative cohorts [298,299,300]; therefore, the applicability of the designed mathematical models is low. Certain brain aging studies, which primarily focus on middle-aged and elderly individuals, often overlook the potential impacts of individual prenatal conditions and childhood trauma on the brain health and BA of the study participants [301]. The application of the concept of AA to localized degeneration presents a challenge, since different brain parts become older unevenly [302]. For example, in localized ND, BA assessment reflects the level of damage to the most vulnerable brain parts (e.g., *substancia nigra* and *nucleus ruber* in PD) [303,304,305]; however, one should also consider the brain resources that can minimize atrophy effects [306]. In systemic ND, the brain ages faster than in localized ND [307,308], and the difference in the pace of atrophic changes is apparent [309].

ND has a multifactorial nature, and contemporary neuroscience currently lacks a comprehensive understanding of how these various factors interact. It is still unclear whether chronic diseases lead to or result from ND [310,311], since genetic, epigenetic, and lifestyle factors interact in an undefined way [312,313]. Several studies have revealed a misalignment between dementia risk, cognitive performance, MBM levels and the impact of medications on study results [314,315,316]. Finally, yet importantly, AA represents a diagnostic, but not pathognomonic, signature in ND and in psychiatric diseases such as schizophrenia, bipolar disorder, and major depressive disorder [295,317,318]. The diverse symptoms seen in these patients cannot be solely attributed to the aging of the brain [295,319,320].

## 6. A Roadmap for the Future of Neuroaging Science

*The statistical criteria and parameters* include sample size, age range, and data normalization. Statistical method selection will improve diagnostic models based on specific MBMs. Studies can benefit from the integration of epigenetics, exploration of additional epigenomic markers of aging, and generation of data in non-human aging models.

*Molecular clocks* could be useful for investigating aging in specific organs and tissues. Organ- and tissue-specific clocks will unravel the complexity of aging in a multicellular biological system. Animal studies have reported some powerful techniques that use the mutation rate of biomolecules to deduce the time [321]. These include organ-specific clocks for the liver [322,323,324], lungs [322,323], blood [323,325], heart and cortex [322], adipose, kidney, muscle tissues [323], and multiple tissues [326].

*Single-cell epigenomics analysis* provides a deep insight into aging [327,328]. For instance, lifetime-dependent cell-to-cell variability in methylation, or so-called “epigenomic noise”, occurs in human immune cells in the blood and in mouse muscle stem cells [329,330]. Epigenomic noise results in increased transcriptional heterogeneity, especially in stem cell niche genes [329]. A recent trend is the construction of epigenetic clocks at a single-cell level by applying novel methods [331,332] and deep-learning computer algorithms [333,334,335].

*New epigenetic marks* of aging are another challenge, and represent interesting opportunities. Links between aging and DNA modifications other than methylation are known, but poorly understood. In mice, the senescence of hippocampus cells deregulates histone H4 acetylation (H4K12) [336] and accumulates histone variant H2A.Z [337]. In the brain of AD patients, researchers found acetylated histones H3 (H3K9ac) and H4 (H4K16ac) [166,338]. Longevity in mammals is linked to histone acetylation by SIRT6 HDAC, and this discovery unlocks the potential for the development of senolytics [339,340,341].

*Distinct aging phenotypes* called “ageotypes” have been identified recently through the longitudinal profiling of multiple omics data. These personalized physiological subsets of aging reflect the impact of various individual factors on the aging rate, which depends on genetics, epigenetic changes, lifestyle habits, and environmental exposure. Models reflecting age will improve diagnostic accuracy as new information is added [98]. By integrating biomarkers of aging into a model using ageotypes, the effectiveness of interventions in each subgroup can be monitored [98,342,343].

*Genetic predispositions* associated with prototypical progeroid syndromes contribute to our knowledge of mechanisms underlying aging. Genome instability disorders resulting from these recessive mutations are categorized into three groups, which include conditions related to the following: sunlight hypersensitivity, such as Xeroderma pigmentosum, Cockayne syndrome, and trichothiodystrophy; disorders associated with ionizing radiation hypersensitivity, including Ataxia telangiectasia and Nijmegen breakage syndrome; and progeroid disorders [344,345,346]. Studies on the aforementioned disorders can also lead to the discovery of anti-aging treatments.

*Unique animal models* used in aging science exhibit the following age-related features: accelerated senescence, damage of the nuclear envelope, and increased accumulation of genomic lesions [347]. Interventions and modulators are commonly tested with well-developed mouse aging models [346]. Mouse models demonstrated epigenetic clock acceleration by a high-fat diet, the effects of rapamycin, and caloric restriction [322,324]. The use of certain established AA models, for example, D-galactose (D-Gal)-administered rodent models, provides a solid basis for the extensive searches and validation of senolytics (Table 4). In these models, D-gal induces AA via the production of reactive oxygen species (ROS) and advanced glycation end-products. D-gal reduction by aldose reductase causes the accumulation of galactitol. Once accumulated, galactitol depletes NADPH, decreases glutathione reductase activity, and acts as a metabotoxin, neurotoxin, and hepatotoxin. Killifish (*Nothobranchius furzeri*) is the vertebrate with the shortest captive lifespan, which makes the species suitable for modeling senescence [348,349,350,351,352,353,354]. Certain animals can mimic aspects of human aging in longevity models, and may provide robust data in aging science, such as the following: naked mole rats (*Heterocephalus glaber*, *Fukomys* mechowii) [355,356,357], Brandt’s bat (*Myotis brandtii*) [358,359,360], olm (*Proteus anguinus*) [361,362,363], bivalves *(Arctica islandica)* [364,365], hydra *(Hydra vulgaris*/*Hydra magnipapillata*) [366,367,368,369], and planaria *(Schmidtea mediterranea*) [370,371,372].

## 7. Conclusions

- Various theories and hypotheses support a paradigm shift in the science of aging. A wealth of data suggests that various processes, influenced by internal and external factors, result in diverse mosaic changes in organisms occurring at different rates, rather than following a uniform, gradual aging pattern.
- The concept of accelerated aging should be considered in the context of personalized characteristics, and methodological limitations should be taken into account. Applying the concept to localized brain neurodegeneration is challenging, since different brain regions and structures age at different rates.
- A healthy lifestyle in a favorable environment, the stimulation of regenerative processes, hygiene, immunization, targeted drug therapy, and balanced metabolism are some of the key approaches that can help slow down brain aging.
- Certain molecular characteristics and substances, including epigenetic changes, differentially expressed genes, and non-coding RNAs, could serve as potential bio-markers and pharmaceutical targets in space biomedicine and may have implications for aging in terrestrial conditions.
- Future research could offer clinics and society new therapeutic possibilities to deal with neuroaging. Studying the connection between space travel and aging in different models and humans can help to improve the safety of space exploration and develop new methods to address neuroaging challenges on Earth.

## 8. Glossary

*Aging* represents the natural time-related deterioration of physiological organ function that is a risk factor for cardiovascular disease, cancer, and neurodegeneration [403].

*Cellular senescence* is a natural defense mechanism preventing abnormal growth and aberrant cell proliferation and contributing to age-related diseases [404]. It is mainly induced by an arrest of the cell cycle in the G1 phase [405].

*Chronological age* is the amount of time elapsed from birth to a given date, and the main way of defining age [406].

*Biological aging* occurs as an organism gradually accumulates damages on the cellular level [407].

*Biological age* is the age corresponding better to the “true life expectancy” of an individual than the chronological age [407].

*Accelerated (pathological) aging* is premature, rapid aging induced by life stressors and associated with telomere shortening [408,409].

*Delayed (decelerated) aging* is an outcome of anti-aging medicine which could slow down but not reverse senescence, delay the onset of degenerative diseases, control their progression, and moderately extend the lifespan [403].

*Brain aging* can be distinguished from aging of other organs as the lifespan of neurons, as postmitotic cells, begins at birth and is equivalent to the lifespan of the entire organism. Although some neurons may die during life, no massive loss of cerebral cells occurs in normal brain aging [410].

*Neurodegenerative diseases* are characterized by the progressive dysfunction of specific populations of neurons, determining clinical picture. Neuronal loss is associated with accumulation of misfolded proteins, the hallmarks of proteinopathies [411].

*Alzheimer’s disease* is characterized by a massive loss of neurons, disintegration of brain regions, and the complete disturbance of brain function accompanied by accumulation of mutations of mitochondrial DNA in neurons [410].

*Dementia* is a cognitive decline of severity sufficient to compromise a person’s daily function [412].

*Mild cognitive impairment* (syn. dementia prodrome, incipient dementia, or isolated memory impairment) is a cognitive decline greater than expected for an individual’s age and education level; however, it does not interfere with the activities of daily life [413]. It is a transitional stage to dementia from normal aging [414].

*Molecular mechanisms of aging*: Aging is associated with an increase in DNA lesions, defects in DNA repair mechanisms, and critically shortened telomeres which trigger an irreversible growth arrest and cellular senescence. In cancer and stem cells, the telomere length is maintained by telomerase [415,416].

The biological clock is an internal alarm clock which adjusts physiology to the varying demands of the solar cycle. Biological clock time is entrained to solar time [417]. A biological rhythm is maintained under constant conditions [418]. Biological clocks reflect the cycles that prevail in life on Earth: tides, day–night, month, and year [419]. Every time the earth orbits the Sun, we become one year older chronologically, yet we know that the rate of biological aging varies greatly [420].

The *genetic clock of biologic rhythms* comprises the intrinsic mechanisms for regulating rhythms from cells to organism, which are embedded into genetic clockwork [419].

The *epigenetic clock* is a biomarker of aging that has been developed using DNA methylation measurements [421]. There is a level of plasticity in gene expression [420], and the ‘DNA methylation age’ provides an accurate estimate of age across tissues and at different stages of life [420].

A *biomarker* is a quantifiable measure that is used as an indicator of responses to treatment or biological or pathological processes. This characteristic may be derived from molecular, histological, radiographical, or physiological data [422].

*Molecular biomarkers of aging* are the molecular targets that capture key aspects of biological age and accurately predict the risk of age-related diseases and mortality. These include markers of cellular senescence, genomic instability, telomere attrition, and mitochondrial dysfunction, among others [421].

*MicroRNAs (miRNAs)* are small endogenous RNAs around 20–25 nucleotides in length that regulate gene expression post-transcriptionally [423,424].

*Circular RNAs* are non-coding RNA molecules with closed-loop secondary structures. They are involved in the regulation of transcription, gene expression, and miRNA functions [425].

*Long non-coding RNAs (lncRNAs)* are untranslated RNA transcripts longer than 200 nucleotides that can regulate the expression of other genes [426,427].

## Figures and Tables

**Table 1:** Top 10 traditional and alternative hypotheses for AD etiology.

	Hypothesis	Evidence	Ref.
1	Aβ cascade	Extracellular deposits of Aβ	[74]
2	Tau	Tau aggregation in NFTs	[75]
3	Inflammation	Pro-inflammatory cytokine secretion in plaque zone	[76,77,78]
4	Excitotoxicity	Excitotoxicity promotes cell death and ND in AD	[79,80]
5	Cholinergic system	Damage of cholinergic neurons associated with dementia	[81,82,83]
6	Dopaminergic system	Decreased levels of dopaminergic neuromediators	[84,85]
7	Oxidative stress	Oxidative stress increases accumulation of Aβ	[86,87,88]
8	APOE/TREM	APOE and TREM2 are significant risk factors of AD	[89,90,91]
9	NV Unit	NVU dysfunction plays crucial role in AD pathogenesis	[92,93]
10	Gut microbiome	Gut microbiome affects neurogenesis, BBB, glia formation	[94,95,96]

**Table 2:** Non-coding RNAs involved in normal brain aging and neurodegeneration.

	ncRNA Class (Acronym)	Molecular Species	Ref.
1	Long non-coding RNA (lncRNA)	17A, GAS5, GDNFOS, BACE1-AS, NAT-Rad18, 51A, HOTAIR, MALAT1, NaPINK1, AS Uchl1	[171,172,173,175,176,177,178]
2	MicroRNA (miRNA)	miR-106a, miR-520c, miR-20a, miR-19, miR-106a/b, miR-101, miR-433, miR-145, miR-375, miR-939, miR-20a, miR-17, miR-147, miR-323-3p, miR-644, miR-153, miR-144, miR-34, over 20 exosomal miRNAs in CSF	[179,180,181,182,183,184,185,186,187]
3	Small interfering RNA (siRNA)	APP-siRNA, siBACE1	[188,189,190]
4	Circular RNA (circRNA)	CIRS-7, cirC_0000400, cirC_0000331, cirC_0000406, cirC_0000798, list of 1167 circRNAs in rat brain	[191,192,193,194]
5	Enhancer RNA (eRNA)	Bdnf-Enhg1, Bdnf-Enhg2, AANCR, Evf2	[195,196,197]
6	Long intergenic non-coding RNA (lincRNA)	BCYRN1, Xist	[198,199,200]
7	Piwi-interacting RNA (piRNA)	piR-hsa-1281, piR-hsa-1280, piR-hsa-1282, piR-hsa-27492, list of 1251 brain piRNAs	[201,202]
8	Y RNA(yRNA)	nELAVL/Y RNA complex, hY1, hY4, hY5	[203,204]

**Table 3:** Non-coding RNAs involved in response to space environment.

Source/Model	ncRNA Molecules/Effects	Ref.
1 Blood plasma of three astronauts	27 differentially expressed exosomal lncRNAs (15 up-regulated, 12 down-regulated)	[262]
2 Datasets from NASA’s GeneLab	13 miRNA which are common in all studies and directly interact with TGF-β1, being the most common regulator of responses to microgravity and/or space radiation	[263]
3 59 astronauts’ data and NASA’s GeneLab datasets	Global transcriptomics analysis and other omics data indicate mitochondrial stress as a consistent phenotype of spaceflight	[264]
4 Rodent serum; immune cells of two astronauts	Shared circulating miRNA signatures observed in both rodents and humans following simulated spaceflight of varying durations. miR-125, miR-16, and let-7a regulate vascular damage caused by simulated deep space radiation	[265]
5 Hindlimb unloaded mouse model and astronauts’ datasets	Circulating plasma microRNAs involved in immune system dysregulation in simulated deep spaceflight rodent model compared with astronauts’ data	[266]
6 Angiogenesis 3D human HUVEC cell culture.	Inhibition of three specific miRNAs, namely miR-16-5p, miR-125b-5p, and let-7a-5p, helped decrease cellular damage caused by exposure to ionizing radiation	[267]
7 Human astrocytes treated with proton radiation (3 Gy)	13 miRNAs significantly down-regulated after exposure to high-energy radiation. hsa-miR-762, let-7c-5p, let-7b-5p regulate genes related to psychological issues and motor and cognitive delays	[268]
8 Proton-irradiated mouse (2 Gy)	14 mouse testis, 8 liver, and 8 brain miRNAs dysregulated after irradiation including miR-409-5p, miR-205, miR-100, miR-501-3p, miR99b, miR-674, and miR-412-5p up-regulated in brain, and miR3076-3p down-regulated	[269]
9 *C. elegans* treated with simulated microgravity	Intestinal linc-2, linc-46, linc-61, and linc-78 up-regulated and linc-13, linc-14, linc-50, and linc-125 down-regulated by simulated microgravity (SMG) treatment	[270]
10 *C. elegans* in space and simulated microgravity	Levels of miR-52, miR-84, and miR-124 found changed in both SMG and space mission. Expression altered for 7 neuromuscular genes (*unc-27, nlp-22, fp-1, egl-5, fp-4, mgl-3, unc-94*)	[271]
11 *C. elegans* in spaceflights	12 miRNAs in 4-day spaceflight regulating 4 DNA repair genes (*ddb-1, Y73F8A.24, T05H10.1, wrn-1*); 4 miRNAs in 8-day spaceflight (cel-miR-58, cel-miR-65, cel-miR-84, and cel-miR238) regulating 2 HR and NHEJ repair genes (*rad-50, him-6*)	[272]
12 *C. elegans* in space and simulated microgravity	126 ncRNAs (mostly snoRNA and lincRNA) induced and 16 ncRNA molecules lost during SMG and for 12 days after. asRNAs *anr-33*, *K12G11.14,* and *ZK822.8* induced, whereas *anr-2*, *anr-9*, and *Y49A3A.6* silenced during and 12 days after SMG	[273]
13 Blood T cells from 8 (10) humans in dry immersion bed	Subset of lncRNAs affected during dry immersion simulated microgravity exposure: HCG11 and LINC00861 up-regulated; MALAT1, PRANCR, DLEU2, CHASERR, PVT1, and PDCD4-AS1 down-regulated in dry immersion	[274]
14 Mouse osteoblasts in simulated microgravity	427 differentially expressed circRNAs identified in osteoblast-differentiating murine MC3T3-E1 cells were exposed to SMG	[275]
15 Datasets from ImmPort SGA and NASA’s GeneLab	Analysis of ImmPort small-for-gestational-age (SGA) fetuses and NASA’s GeneLab murine datasets for hindlimb unloading SMG model; simulated Galactic Cosmic Radiation (GCR) beam at 0.5 Gy; simulated Solar Particle Event (SPE) radiation at 1 Gy identified 13 miRNAs involved in potential SGA risk during spaceflight	[276]
16 Rat simulated microgravity in SCSE model	miR-455-3p, miR-206-3p, miR-132-3p, and miR-16-5p observed to be increased in response to depressive behavior induced by simulated spaceflight	[277]

**Table 4:** Recent studies on D-Gal AA models and effects.

	Model	Effects	Ref.
1	Senescent Kupffer cells in mice	MicroRNA-7 deficiency ameliorated d-galactose-induced aging. miR-7 deficiency reduced IL-1β in liver tissue, and the inhibition of IL-1β*in vivo* slowed down aging in mice. KLF4 was found to be down-regulated in senescent Kupffer cells.	[373]
2	D-gal liver aging model	Multiple pharmacological agents were used to reverse D-gal-induced liver aging.	[374]
3	CBM mice cells	In the CBM of the D-gal groups, the transmembrane potential dropped and the ATP level decreased while the level of β-galactosidase increased.	[375]
4	D-gal human erythrocytes	D-gal led to Hb glycation, produced substantial changes in the endogenous antioxidant system, and induced early aging in human erythrocytes.	[376]
5	OLETF rat D-gal aging model for AD	Levels of p-IRS1, p-IRS2, IDE, and p-GSK3β significantly elevated, while p-PI3K-p85*α*, and p-Akt decreased. Electroacupuncture enhanced cognitive function and alleviated insulin resistance.	[377]
6	DHP application to D-gal mouse aging model	*D. huoshanense* polysaccharide (DHP) protected the antioxidant enzymes SOD, GSH-PX, and CAT from excessive ROS, blocked the P53/P21 signaling pathway, and showed a potential neuroprotective effect on D-gal-mediated cognitive disorders.	[378]
7	D-gal-induced mouse aging model and senescent cells	Gliclazide regulated neuronal apoptosis in the aging mouse model and in D-gal-induced senescent cells, showed a beneficial effect on D-gal-induced neuronal injury, and was selected as a candidate drug for inhibiting age-related mental decline.	[379]
8	Hippocampus cells of D-gal mouse aging model	Phlorizin increased antioxidant enzyme activity, showed anti-inflammation effects by regulating the IL-1β/NF-kB pathways in the brain, and alleviated neuroapoptosis via Bax, Bcl-2, and caspase 3. Phlorizin was suggested as a potential anti-aging drug.	[380]
9	D-gal-induced rat aging model	Folic acid partially reversed D-gal-caused oxidative damage to lipids and protein in the hippocampus and prefrontal cortex in the D-gal-induced rat aging model.	[381]
10	D-gal aging rat model and senescent PC12 cells	A combination of lycopene and β-NMN slowed down aging more efficiently than monotherapy. The combination down-regulated the senescence-related *p53*, *p21*, and *p16* genes and increased Nrf2 signaling in aging models.	[382]
11	D-gal-induced mouse brain aging model	Dihydromyricetin from *Ampelopsis grossedentata* showed strong neuroprotective effects, improved spatial cognition, and inhibited lipid peroxidation, malondialdehyde (MDA), AGE production, and *p53, p21*, and *p16* gene expression.	[383]
12	PSP application to D-gal-induced rat aging model	The polysaccharide of *Polygonatum sibiricum* (PSP) significantly improved learning and reversed the kidneys’ pathological changes. PSP up-regulated *Klotho* and down-regulated *FOXO3a* in renal tissue and the femoral expression of FGF-23 protein.	[384]
13	D-gal-induced mouse brain aging model	Saponin (ginsenoside) Rg2 from *Panax ginseng* delayed brain aging by restoring D-gal-induced impaired memory function and redox system balance in mice.	[385]
14	SDE application to D-gal-induced mouse aging model	Skeels fruit extract (SDE) reduced acetylcholinesterase activity in the brain, iNOS activity in serum, activated SOD and glutathione in the liver and brain, inhibited *TNFα, NF-kB, IL-1β, IL-6*, and *p53*, and induced *SIRT1* and *Klotho* in the brain and liver.	[386]
15	Vit D application to D-gal-induced rat aging model	Vitamin D (Vit D) improved cardiac hypertrophy, elevated cardiac mitophagy, and reduced apoptosis.	[387]
16	D-gal-induced mice hepatocellular aging model	In the D-Gal/melatonin co-treated group, melatonin treatment alleviated D-Gal-induced hepatocyte impairment and reduced the expression of inflammatory genes *IL1-β*, *NF-κB*, *IL-6*, *TNFα,* and iNOS.	[388]
17	Aging mice, D-gal-induced mouse aging model, human HK-2 cells	Methyltransferase-like protein 3 (METTL3) helped N6-methyladenosine (*m*6A) modification involved in morbid changes. miR-181a-5p counteracted HK-2 senescence by targeting the NF-*κ*B pathway. METTL3 promoted the maturation of miR-181a-5p and inhibited the expression of *NF-κB* and *IL-1α*.	[389]
18	D-gal-induced mouse aging model	Metrnl expression significantly increased in the hippocampus. Metrnl knockout aggravated cognitive impairment and reduced the levels of hippocampal BDNF, TrkB, and glial fibrillary acidic protein. Metrnl regulated cognitive functions in aging, and it was considered for the treatment of aging-related cognitive dysfunction.	[390]
19	D-gal-induced mouse aging model	An ethyl acetate fraction of *Physalis alkekengi* (PAE) decreased the activity of senescence-associated *β*-galactosidase in the liver, spleen, and hippocampus and oxidative stress in the liver, plasma, and brain. It can be used to prevent or treat aging-associated disorders.	[391]
20	Calcium dobesilate (CaD) application to D-gal-induced mouse aging model	Calcium dobesilate (CaD) reversed the body weight loss and cognitive impairment of D-gal-treated animals. CaD inhibited oxidative stress in the brain by decreasing the MDA level and increasing activity of SOD, glutathione peroxidase (GPx), and catalase (CAT). CaD was considered as a candidate drug against cognitive impairment in aging.	[392]
21	TQ and Cur application to D-gal-induced rat aging model	Thymoquinone (TQ) and curcumin (Cur) suppressed D-gal-induced aging in the brain and heart. The TQ and Cur combination reduced necrosis in the brain and heart by D-gal, the levels of brain caspase 3, BCL2, calbindin, heart caspase 3, and calcium-binding adapter molecule 1, inhibited *p53*, *p21*, *Bax*, and *CASP-3* expression, and may prevent aging.	[393]
22	D-gal-induced mouse aging model, and hBM-MS cells	A bicyclic monoterpenoid camphorquinone (CQ) reduced senescence in mouse heart tissues and human bone marrow mesenchymal stem cells (hBM-MSCs). In both models, CQ boosted AMPK/SIRT1 activation and autophagy.	[394]
23	AN application to D-gal-induced mouse aging model	*Schisandra sphenanthera* has been used in traditional Chinese medicine for thousands of years. The study reported the immunomodulatory activity of a monomer of *S. sphenanthera* lignans (Anwulignan, AN) in aging.	[395]
24	PL 1-3 application to D-gal-induced mouse aging model	A derivative from Piperlongumine (PL 1-3) decreased the antioxidative stress in the serum, liver, kidney, and brain of aging mice. PL 1-3 up-regulated the expression of sirtuin 1 and down-regulated the expressions of *p53*, *p21*, and *p16* genes. It also reversed damages induced by D-gal in the liver, kidney, and spleen.	[396]
25	MLE application to *C. elegans* and D-gal-induced mouse aging model	Mulberry leaf extract (MLE) significantly prolonged the average life span of *C. elegans*. In the mice model, MLE protected against oxidative stress and ameliorated the decreased body weight and organ index. MLE up-regulated total SOD and total antioxidant capacity. It activated the MPK/SIRT1/PGC-1*α* pathways and reduced ROS and MDA levels.	[397]
26	D-gal-induced Wistar rat aging model	In high-fat diet-fed rats, D-gal-induced aging elevated AGEs, significantly impaired bone microarchitecture, and increased bone inflammation and resorption. In obesity, D-gal-induced aging aggravated bone dyshomeostasis in a time-dependent manner.	[398]
27	T37a application to D-gal-induced mouse aging model	*Bifidobacterium longum* T37a decreased the spleen and liver index, increased HDL-C, decreased LDL-C and MDA levels in the liver, showed antioxidant properties in the DPPH assay and anti-lipid peroxidation test, and was considered as a candidate anti-aging drug.	[399]
28	GNL application to D-gal-induced mouse aging model	Geraniol (GNL) induced a significant increase in learning and memory, up-regulated *Nrf2* and *HO-1*, and reduced oxidative stress and apoptosis. Therefore, GNL was suggested as a promising agent for treating neuroinflammation-induced cognitive impairment.	[400]
29	D-gal-induced mouse aging model	Recombinant IL-33 elevated osteogenic parameters, reduced senescence markers, and exerted neuroprotective potential in osteoblasts in an aging mice model. IL-33 can be considered as a therapy for the treatment of aging-induced bone loss and memory impairment.	[401]
30	QUE application to D-gal-induced Wistar rat aging model	Quercetin (QUE*)* potentially attenuated oxidative alterations of the pancreas and kidneys, reduced the levels of apoptotic and inflammatory markers, and up-regulated antiapoptotic, proliferative, antioxidant, and functional markers. QUE is considered as a promising natural protective compound that could be used to delay aging.	[402]

## Abbreviations

The following abbreviations are used in this manuscript:

5-mC	5-methylcytosine
AA	accelerated aging
AAG	gene related to accelerated aging
AD	Alzheimer’s disease
asRNA	antisense RNA
BA	biological age
BBB	blood–brain barrier
BM	biomarker
circRNA	circular RNA
DA	decelerated aging
D-gal	D-galactose
eRNA	enhancer RNA
GCR	Galactic Cosmic Radiation
HC	healthy control
LEO	low Earth orbit
lncRNA	long non-coding RNA
lincRNA	long intergenic non-coding RNA
MBM	molecular biomarker
MCI	mild cognitive impairment
MG	microgravity
miRNA	microRNA
ncRNA	non-coding RNA
NA	normal aging
ND	neurodegeneration
NDG	gene that predisposes neurodegeneration
NV	neurovascular
NVU	neurovascular unit
PD	Parkinson’s disease
piRNA	Piwi-interacting RNA
ROS	reactive oxygen species
siRNA	small interfering RNA
snoRNA	small nucleolar RNA
SMG	simulated microgravity
SMS	space motion sickness
SPE	Solar Particle Event
Xist	X inactivation-specific transcript
yRNA	Y RNA
